# Reduction of In Vivo Placental Amino Acid Transport Precedes the Development of Intrauterine Growth Restriction in the Non-Human Primate

**DOI:** 10.3390/nu13082892

**Published:** 2021-08-23

**Authors:** Fredrick J. Rosario, Anita Kramer, Cun Li, Henry L. Galan, Theresa L. Powell, Peter W. Nathanielsz, Thomas Jansson

**Affiliations:** 1 Division of Reproductive Sciences, Department of Obstetrics and Gynecology, University of Colorado Anschutz Medical Campus, Aurora, CO 80045, USA; anita.kramer@cuanschutz.edu (A.K.); Theresa.Powell@cuanschutz.edu (T.L.P.); thomas.jansson@ucdenver.edu (T.J.); 2 Texas Pregnancy and Life-Course Health Center, Department of Animal Sciences, University of Wyoming, Laramie, WY 82071, USA; cli5@uwyo.edu (C.L.); Peter.Nathanielsz@uwyo.edu (P.W.N.); 3 Southwest National Primate Research Center, Texas Biomedical Research Institute, San Antonio, TX 78227, USA; 4 Division of Maternal-Fetal Medicine, Department of Obstetrics and Gynecology, University of Colorado Anschutz Medical Campus, Aurora, CO 80045, USA; henry.galan@cuanschutz.edu; 5 Section of Neonatology, Department of Pediatrics, University of Colorado Anschutz Medical Campus, Aurora, CO 80045, USA

**Keywords:** maternal-fetal exchange, amino acids, neutral, trophoblast, fetal growth restriction

## Abstract

Intrauterine growth restriction (IUGR) is associated with reduced placental amino acid transport (AAT). However, it remains to be established if changes in AAT contribute to restricted fetal growth. We hypothesized that reduced in vivo placental AAT precedes the development of IUGR in baboons with maternal nutrient restriction (MNR). Baboons were fed either a control (ad libitum) or MNR diet (70% of control diet) from gestational day (GD) 30. At GD 140, in vivo transplacental AA transport was measured by infusing nine ^(13)^C- or ^(2)^H-labeled essential amino acids (EAAs) as a bolus into the maternal circulation at cesarean section. A fetal vein-to-maternal artery mole percent excess ratio for each EAA was measured. Microvillous plasma membrane (MVM) system A and system L transport activity were determined. Fetal and placental weights were not significantly different between MNR and control. In vivo, the fetal vein-to-maternal artery mole percent excess ratio was significantly decreased for tryptophan in MNR. MVM system A and system L activity was markedly reduced in MNR. Reduction of in vivo placental amino acid transport precedes fetal growth restriction in the non-human primate, suggesting that reduced placental amino acid transfer may contribute to IUGR.

## 1. Introduction

Intrauterine growth restriction (IUGR) affects about 30 million babies each year worldwide and is a significant cause of perinatal morbidity and mortality [1,2,3]. In addition, IUGR is also linked to an increased risk of developing cardiovascular dysfunction, insulin resistance, and other metabolic disorders in adulthood [4,5,6]. In the developed world, the failure of the normal increase in uteroplacental blood flow is believed to be the most common etiology of IUGR. In contrast, maternal undernutrition remains the leading cause of in utero restricted growth [7] in developing regions. Furthermore, more than 50 million Americans live in households experiencing food insecurity or hunger sometime during the year [8]. Therefore, exploring the effects of maternal undernutrition on placental function is highly relevant not only to many parts of the world where inadequate food intake in pregnant women is still a significant concern. Moreover, different etiologies of IUGR are associated with strikingly similar changes in placental signaling and function, including downregulation of insulin/IGF-1 and mTOR signaling and decreased amino acid transport capacity [7,9,10,11,12]. Thus, it is possible that studies of IUGR due to nutrient restriction to mother may also increase our understanding of IUGR due to compromised uteroplacental blood flow.

The syncytiotrophoblast, the transporting epithelium of the human placenta, mediates the transport of nutrients from the maternal to the fetal circulation. The System L amino acid transporter is a sodium-independent exchanger. It mediates cellular uptake of essential amino acids, consists of branched-chain (such as L-leucine) and neutral aromatic amino acids (including L-phenylalanine) [13]. The functional System L transporter is a heterodimer, consisting of a light chain (typically LAT1, SLC7A5 or LAT2, SLC7A8) covalently attached to a heavy chain (4F2hc/CD98; 4F2 cell-surface antigen heavy chain/cluster of differentiation 98, SLC3A2) [14]. Both LAT1 and LAT2 transporters contribute to trophoblast system L transport capacity [15].

System A catalyzes the sodium-dependent net uptake of non-essential neutral amino acids into the cell [16]. All three isoforms of System A (SNAT1, SLC38A1; SNAT2, SLC38A2 and SNAT4, SLC38A4) are expressed in the human placenta. Trophoblast-specific SNAT2 or SNAT4 gene knockout studies indicate that placental system A amino acid transport activity is critical to placental and fetal growth in mice [17]. Furthermore, placental system A and L amino acid transport activity is decreased in human IUGR, suggesting that changes in placental amino acid transport activity may directly contribute to fetal growth restriction [18,19,20,21,22,23]. However, the mechanistic link between changes in placental amino acid transport capacity and the development of IUGR in women is unknown. This information is needed to better understand the pathophysiology of IUGR and future efforts to develop interventions to improve placental function and alleviate restricted fetal growth. Importantly, this question cannot be easily addressed in human pregnancy.

In rats, we demonstrated that maternal protein restriction causes down-regulation of placental amino acid transport several days before fetal size reductions were observed [9,12]. However, the rodent placenta is very different from the human placenta, and it is not entirely clear if this information can be extrapolated to women. Given the striking similarities in placental structure and the close evolutionary relationship to humans, studies using non-human primate models are likely more informative. We have developed a baboon model of 30% global caloric maternal nutrient restriction (MNR), which results in IUGR, reduced fetal circulating levels of essential amino acids, and structural and functional changes in a range of fetal organs [24,25,26,27,28,29,30,31,32,33] and long term increased risk for poor health.

Using this model, we reported that at gestational day (GD) 165, which is approximately 90% of gestation (term~GD 184), MNR was linked to decreased placental amino acid transport and IUGR [11]. Similarly, we demonstrated that System A amino acid transporters activity decreased in isolated syncytiotrophoblast microvillous plasma membranes at GD 120 (~65% of gestation) [34]; at this point in gestation, there was no reduction in fetal size [34]. Here, using control and MNR baboons at GD 140, we tested the hypothesis that decreased in vivo placental amino acid transport precedes the development of IUGR in baboons with MNR (maternal nutrient restriction).

## 2. Materials and Methods

### 2.1. Animal Maintenance

All experimental animal protocols were subject to ethical review and approved by the TBRI (Texas Biomedical Research Institute) Institutional Animal Care and Use of Laboratory Animals at San Antonio, Texas. In addition, all the experiments utilizing Baboons (*Pepion species*) were carried out in TBRI primate research facilities authorized by the AAALAC (Association for the Assessment and Accreditation of Laboratory Animal Care).

### 2.2. Feeding

Morphometric assessments were done before pregnancy to guarantee all the baboons used in the current experiment were homogenous in bodyweight and general morphometrics. We used an individual feeding system. Briefly, each baboon was fed individually. The feeding times were 07.00 a.m.–09.00 a.m. or 11.00 a.m.–1.00 p.m., allowing careful monitoring and control of individual diets in each feeding cage. Both control and experimental group baboons had access to water ad libitum in each feeding cage. During feeding time, each baboon’s weight was measured with the help of a digital weighing system (GSE 665; GSE Scale Systems, Allen Park, MI, USA).

### 2.3. Composition of Diet

Purina Monkey biscuits (Cat # 5038) were purchased from Purina (St. Louis, MO, USA). Each biscuit’s dietary composition was ≤6% crude fiber, ≥15% crude protein, ≥5% crude fat, 5% ash, ≤3% added minerals, solubilized vitamin C, and other essential vitamins.

### 2.4. Experimental Design

Until 30 days of gestation (GD), all the pregnant baboons had ad libitum access to diet. From GD 30 onwards, MNR group baboons were fed 70% of the feed consumed by the control group, based on a weight adjustment throughout the study period. However, the control group (CTR) baboons continued to feed ad libitum throughout the study period. As previously described, each animal’s daily food intake, mean body weight, and health status were recorded [11].

### 2.5. Blood and Tissue Collection

As described elsewhere [11], at GD 140, baboons were administered ketamine hydrochloride (Dosage: 10 mg/kg), intubated, and anesthetized with the use of isoflurane (starting rate 2% with oxygen, 2 L/min). After that, a cesarean section was performed using standard sterile techniques. Blood samples were collected from the maternal uterine vein and fetal umbilical vein at the cesarean section [34]. Fetuses and placentas were weighed. As described in detail below, villous trophoblast tissue was collected from the placenta, and collected villus tissue was used to isolate syncytiotrophoblast plasma membranes. Adequate postoperative analgesia was administered to baboons (buprenorphine 0.015 mg·kg^−1^·day^−1^ as two doses for three days) following the cesarean section.

### 2.6. Stable Isotope-Labeled Essential Amino Acids

At GD 140, during the time of the cesarean section, a bolus mixture consisting of nine stable isotope-labeled (^13^C or ^2^H) essential amino acids (EAAs) was infused into a maternal peripheral vein over 2 min as described for pregnant women [35], with slight modifications (discussed detail in supplemental methods). The infusate was diluted to 10 mL with sterile saline solution (isotonic), and its composition is listed in Table 1.

### 2.7. Placental MVM and BM Vesicles Preparation

Placentas were collected at cesarean section and washed immediately using ice-cold phosphate-buffered saline (PBS) to clean off excessive blood clots. Chorionic villi were dissected from the placenta into small pieces (roughly 1 cm^3^) and homogenized in ice-cold Buffer D (Composition of Buffer D; 250 mM sucrose, 10 mM HEPES-Tris, and 1 mM ethylenediaminetetraacetic acid, pH 7.4, at 4 °C), containing inhibitors of protease and phosphatase. Syncytiotrophoblast maternal facing microvillous plasma membranes (MVM) and fetal facing basal plasma membranes (BM) were isolated from each placental homogenate, according to a previously published protocol (described in detail in supplemental methods) with minor modifications [36,37].

### 2.8. System A and System L Amino Acid Transport Activity

Briefly, vesicles were preloaded with ice-cold vesicles loading buffer (Biochemical composition: 300 mmol/L mannitol and 10 mmol/L HEPES-Tris, pH 7.4) and incubated overnight at 4 °C. Next, vesicles were centrifuged, and the resulting pellet was resuspended in the same ice-cold vesicle buffer to a final protein concentration of approximately 6 mg of protein/mL of vesicle loading buffer. Finally, vesicles were kept on ice until analysis, which is described in detail in supplemental methods.

### 2.9. Western Blot Analysis

Next, western blot analysis was performed to measure the MVM protein expression of various transporters isoforms [SNAT-2, LAT-1, LAT-2, glucose transporter (GLUT)-1, and taurine transporter (TAUT)] as reported previously [11] (details provided in supplemental methods). Finally, blots were re-probed (after stripping) for β-actin as a loading control.

### 2.10. Statistical Analysis

The number of experiments (n) in each group indicates how many numbers of placenta were analyzed per group. Data are given as means ± S.E.M or Medians ± IQR (Interquartile range). Differences between two independent groups (Control vs. MNR) were tested statistically using the Mann–Whitney test. Using the Graph Pad Prism 5 software, linear relationships between variables of the control and experimental group were assessed using the following analysis (i) bivariate and (ii) Pearson’s correlation coefficients analysis. A *p* value less than 0.05 indicates that the results were statistically significant. The Kolmogorov–Smirnov normality test was performed using the Graph Pad Prism 5 software to determine whether control and MNR group data were normally distributed or not.

## 3. Results

### 3.1. Fetal and Placental Weights of Control and MNR Group

**Figure 1:**
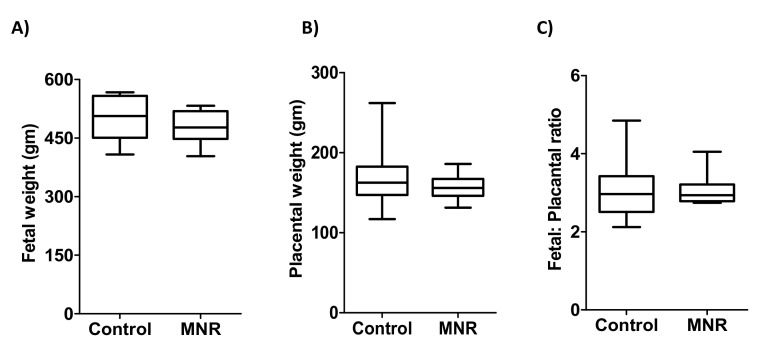
Fetal weight, placental weight and fetal: placental ratio of control and MNR group at GD 140. (**A**) Fetal (Birth) weight (**B**) placental weight and (**C**) fetal: placental weight ratio at gestational day 140 in the control and MNR groups. Medians ± IQR, *n* = 10 control; *n* = 12 MNR. KS normality test *p* value: Fetal weight (Control, *p* > 0.10; MNR, *p* > 0.10; passed normality), Placental weights (Control, *p* > 0.10; MNR, *p* > 0.10; passed normality), Fetal: Placental ratio Control, *p* > 0.10; MNR, *p* > 0.10; passed normality).

Fetal (birth weight) and placental weights were recorded at the time of cesarean section. As shown in Figure 1, there is no statistical difference in fetal weight or placental weight between the control and MNR groups. Furthermore, fetal: placental ratios were identical between control (n = 10) and MNR (n = 12) group animals (Figure 1).

### 3.2. Maternal Enrichment

**Figure 2:**
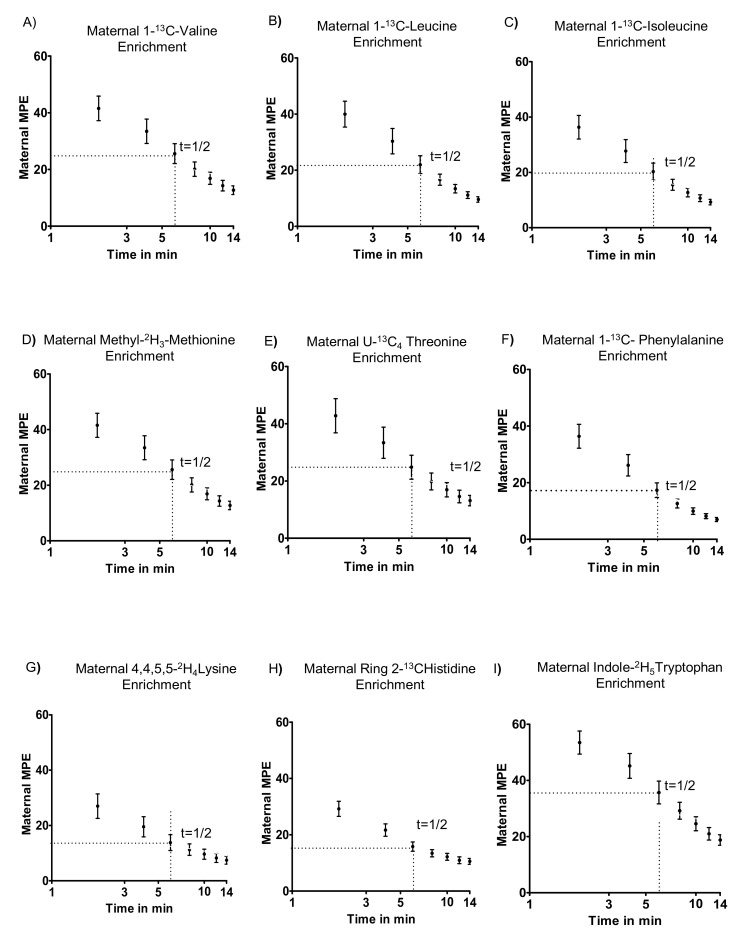
Maternal plasma MPE (mole percent excess) after bolus infusion in control and MNR baboons. (**A**–**I**): MPE of stable isotope-labeled (1-^13^C) valine, (1-^13^C) leucine, (1-^13^C) isoleucine, [Methyl-^2^H_3_] methionine, [U-^13^C_4_] threonine, [1-^13^C] phenylalanine, [4,4,5,5-^2^H_4_] lysine, [Ring 2-^13^C] histidine, and [Indole-^2^H_5_] tryptophan in maternal arterial blood after intravenous infusion of a mixture of labeled essential amino acids to the pregnant baboon at GD 140 (Control, *n* = 8; MNR, *n* = 8). Medians ± IQR, t = 1/2: half-life. Each data point represents combined maternal MPE values of both control and MNR group (*n* = 16 each time point, (Control, *n* = 8; MNR, *n* = 8)).

The maternal plasma enrichment over time was similar between control and MNR groups (data not shown), and data from the two groups (n = 8/each group) is merged in Figure 2. In baboons at GD 140, (1-^13^C) valine, (1-^13^C) leucine, (1-^13^C) isoleucine, [Methyl-^2^H_3_] methionine, [U-^13^C_4_] threonine, [1-^13^C] phenylalanine, [4,4,5,5-^2^H_4_] lysine, [Ring 2-^13^C] histidine, and [Indole-^2^H_5_] tryptophan were rapidly cleared from the maternal circulation. There were no statistically significant differences in the clearance rates among the nine essential amino acids that were analyzed.

### 3.3. Fv/M MPE Ratios

**Figure 3:**
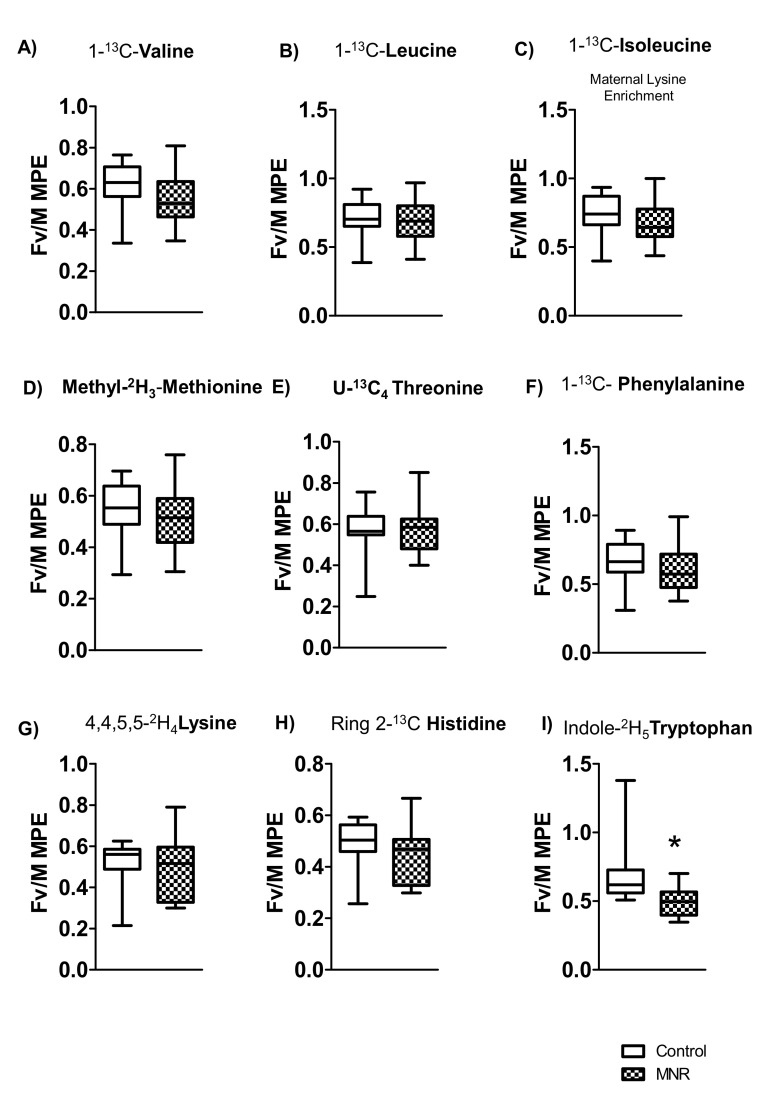
In vivo transplacental transport of (1-^13^C) valine, (1-^13^C) leucine, (1-^13^C) isoleucine, [Methyl-^2^H_3_] methionine, [U-^13^C_4_] threonine, [1-^13^C] phenylalanine, [4,4,5,5-^2^H_4_] lysine, [Ring 2-^13^C] histidine, and [Indole-^2^H_5_] tryptophan. Fetal vein/maternal artery (Fv/M) mole percent excess (MPE) ratios of stable isotope-labeled nine EAAs after intravenous infusion of a mixture of labeled EAAs to the control and MNR pregnant baboon at cesarean section (GD 140). Umbilical vein blood samples were obtained 5 min after the completion of EAA infusion. Fv/M MPE ratios of valine, leucine, isoleucine, methionine, threonine, phenylalanine, lysine, histidine, tryptophan in control (n = 8) and MNR (n = 11) baboons at GD 140. Medians ± IQR, * *p* < 0.05. KS normality test *p* value: (1-^13^C) valine (Control, *p* > 0.10; MNR, *p* > 0.10; passed normality), (1-^13^C) leucine (Control, *p* > 0.10; MNR, *p* > 0.10; passed normality), (1-^13^C) isoleucine (Control, *p* > 0.10; MNR, *p* > 0.10; passed normality), [Methyl-^2^H_3_] methionine (Control, *p* > 0.10; MNR, *p* > 0.10; passed normality), [U-^13^C_4_] threonine (Control, *p* = 0.010, did not pass normality; MNR, *p* > 0.10; passed normality), [1-^13^C] phenylalanine (Control, *p* > 0.10; MNR, *p* > 0.10; passed normality), [4,4,5,5-^2^H_4_] lysine (Control, *p* > 0.10; MNR, *p* > 0.10; passed normality), [Ring 2-^13^C] histidine (Control, *p* = 0.04, did not pass normality; MNR, *p* > 0.10; passed normality), [Indole-^2^H_5_] tryptophan (Control, *p* = 0.01, did not pass normality; MNR, *p* > 0.10; passed normality).

Fetal vein/maternal artery (Fv/M) mole percent excess (MPE) ratios were measured for the (1-^13^C) valine, (1-^13^C) leucine, (1-^13^C) isoleucine, [Methyl-^2^H_3_] methionine, [U-^13^C_4_] threonine, [1-^13^C] phenylalanine, [4,4,5,5-^2^H_4_] lysine, [Ring 2-^13^C] histidine, and [Indole-^2^H_5_] tryptophan that were infused in control (n = 8) and MNR (n = 11) group pregnant baboons at cesarean section. At GD 140, Fv/M MPE ratios for (1-^13^C) valine, (1-^13^C) leucine, (1-^13^C) isoleucine, [Methyl-^2^H_3_] methionine, [U-^13^C_4_] threonine, [1-^13^C] phenylalanine, [4,4,5,5-^2^H_4_] lysine, and [Ring 2-^13^C] histidine were not statistically different among control and experimental (MNR) groups. However, the Fv/M MPE level of the EAAs [Indole-^2^H_5_] tryptophan (*p* = 0.02) was significantly decreased in the MNR group compared with controls (Figure 3).

**Figure 4:**
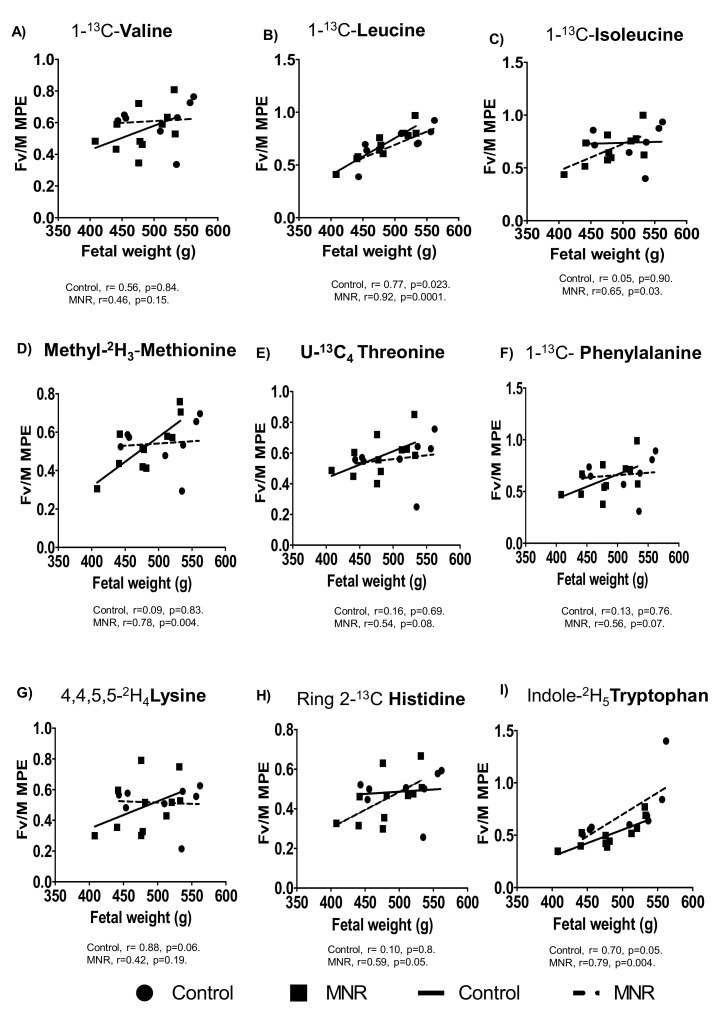
Relationship between Fv/M MPE ratio and fetal weight. (**A**–**I**) Correlation between fetal vein/maternal artery (Fv/M) mole percent excess (MPE) ratio of (1-^13^C) valine, (1-^13^C) leucine, (1-^13^C) isoleucine, [Methyl-^2^H_3_] methionine, [U-^13^C_4_] threonine, [1-^13^C] phenylalanine, [4,4,5,5-^2^H_4_] lysine, [Ring 2-^13^C] histidine, and [Indole-^2^H_5_] tryptophan and fetal weight in control (*n* = 8) and MNR (*n* = 11) baboons at GD140. Pearson correlation coefficient (r).

There were no significant differences in Fv/M MPE ratios for (1-^13^C) isoleucine, [Methyl-^2^H_3_] methionine, and [Ring 2-^13^C] histidine between control (AGA) and MNR (IUGR) fetuses (Figure 3). However, the Fv/MPE enrichment ratio of (1-^13^C) isoleucine, [Methyl-^2^H_3_] methionine, and [Ring 2-^13^C] histidine was positively correlated with fetal weight in the MNR group baboons (Figure 4). Moreover, the Fv/M enrichment ratio of (1-^13^C) leucine and [Indole-^2^H_5_] tryptophan were significantly associated with fetal weight in control and MNR pregnancies (Figure 4).

### 3.4. Effect of MNR on System A and System L Amino Acid Transport Activity in MVM

**Figure 5:**
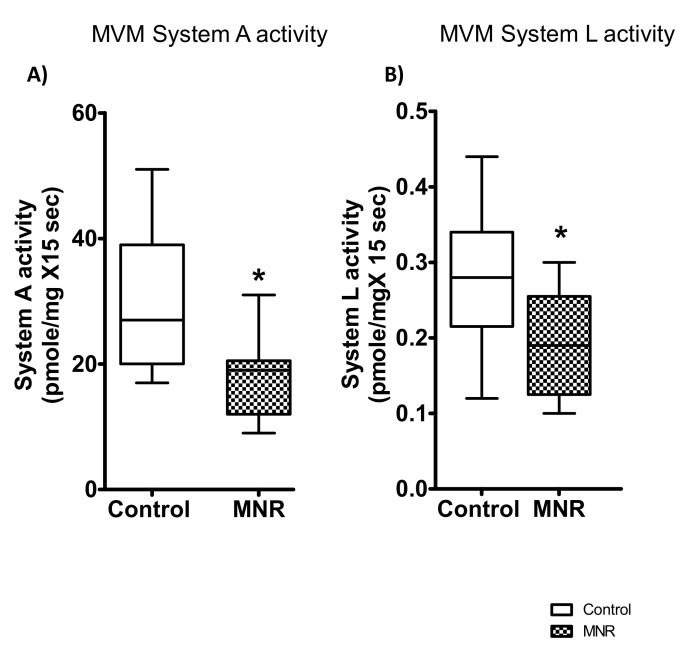
MVM System A and L transport activity. (**A**,**B**) Microvillous plasma membrane vesicles (MVM) system A and system L activity in the MNR and control group. (**A**) System A mediated MeAIB uptake in MVM isolated from control and MNR placentas of baboons at GD 140. (**B**) System L mediated L-leucine uptake in MVM isolated from control and MNR placentas of baboons at GD 140. MVM system A and L uptake in Control and MNR groups were compared using the Mann–Whitney test (*n* = 9/each group, * *p* < 0.05, mean ± S.E.M.). Medians ± IQR, *n* = 10 control; *n* = 12 MNR. KS normality test *p*-value: MVM system A activity (Control, *p* > 0.10; MNR, *p* > 0.10; passed normality), MVM system L activity (Control, *p* > 0.10; MNR, *p* > 0.10; passed normality).

MVM system A-mediated transport activity was reduced by 40% in the MNR group compared to the controls (*n* = 9/each group, *p* = 0.02) (Figure 5). Similarly, mediated uptake of L-Leucine, previously shown to closely represent System L activity in MVM [34,38], was significantly decreased by 30% in the MNR group (*n* = 9/each group, *p* = 0.04, Figure 5).

We determined the relation between System A and System L amino acid transport activity between control and MNR groups. As shown in Supplemental Figure S1, MVM system A activity was positively correlated to system L amino acid transporter activity in the control and MNR groups (Supplemental Figure S1).

**Figure 6:**
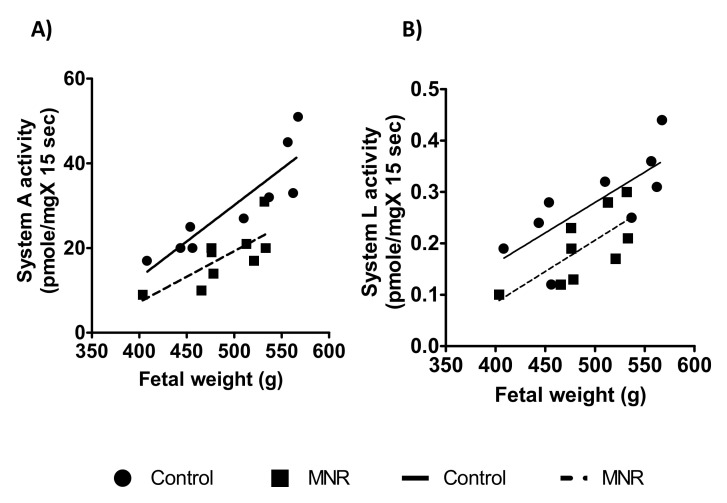
Relationship between MVM System A /system L activity and fetal weight at GD 140 (**A**,**B**). MVM system A/System L activity was positively correlated to fetal weight in both control and MNR groups (System A activity: control, r = 0.88, *p* = 0.0016; MNR, r = 0.75, *p* = 0.01, *n* = 9 in each group; System L activity: control, r = 0.75, *p* = 0.01; MNR, r = 0.72, *p* = 0.03, *n* = 9 in each group, r = Pearson correlation coefficient).

Next, we observed that fetal weight was positively correlated with MVM system A (Figure 6) and System L activity (Figure 6) in the control and MNR groups. However, we did not find a correlation between placental weight and MVM system A or System L amino acid transport activity in the control and MNR groups (data not shown).

### 3.5. Leucine Transport in BM

**Figure 7:**
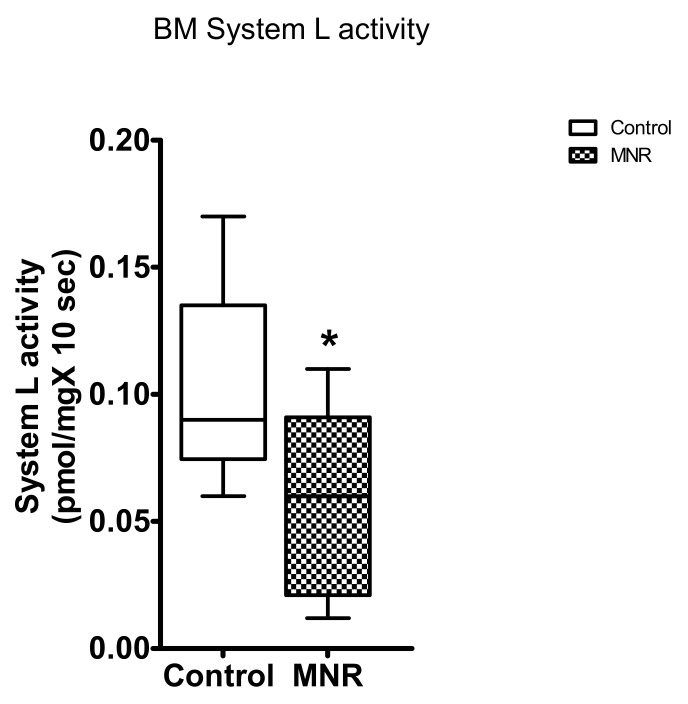
Basal plasma membrane (BM) leucine transport in control and MNR group at GD 140. System L involved L-leucine uptake into BM vesicles isolated from placentas of control and MNR group. BM leucine transport in Control and MNR groups was compared using the Mann–Whitney test (*n* = 9/each group, * *p* < 0.05, Medians ± IQR). KS normality test *p*-value: BM system L activity (Control, *p* > 0.10; MNR, *p* > 0.10; passed normality).

BM mediated L-leucine uptake was significantly decreased by 40% in baboons subjected to MNR compared to the control group (n = 9/each group, *p* = 0.04; Figure 7) at GD 140.

### 3.6. Protein Expression of System L Acid Transporter Isoforms in MVM

**Figure 8:**
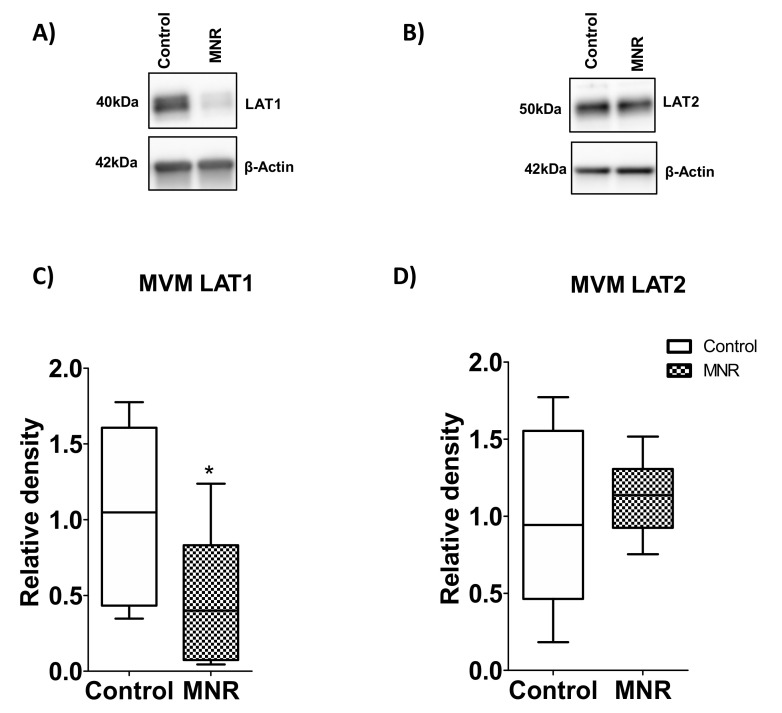
(**C**,**D**) MVM LAT1 and 2 protein expression. Western blot analysis of (**A**) LAT1 and (**B**) LAT2 (system L amino acid transporter isoforms) in MVM isolated from control (**C**) and MNR baboons at GD 140. The histogram shows protein expression data of MVM LAT1 and LAT2. Equal sample loading was performed. After normalization of MVM LAT1 or LAT2 expression to loading control (β-actin), the average density of the control sample band was designated an arbitrary value of 1. Values are given as Medians ± IQR. * *p* < 0.05, Mann–Whitney test (n = 9/each group). KS normality test *p*-value: MVM LAT1 (Control, *p* > 0.10; MNR, *p* > 0.10; passed normality), MVM LAT2 (Control, *p* > 0.10; MNR, *p* > 0.10; passed normality).

Using Western Blot, LAT1 and 2 (system L amino acid transporter isoforms) protein expressions were determined in MVM vesicles isolated from placentas of controls and MNR baboons at GD 140. MVM LAT1 protein expression was significantly lowered by 53% in MNR compared to control group baboons (n = 9/each group, *p* = 0.04, Figure 8A,C). However, MVM LAT2 protein expression was comparable between the controls and MNR (n = 9/each group, *p* = 0.54, Figure 8B,D).

MVM LAT1 expression positively correlated with MVM LAT2 expression in the control group. However, there was no association between MVM LAT1 expression and MVM LAT2 expression in the MNR group (Supplemental Figure S2).

### 3.7. Protein Expression of SNAT2 Transporter Isoform in MVM

**Figure 9:**
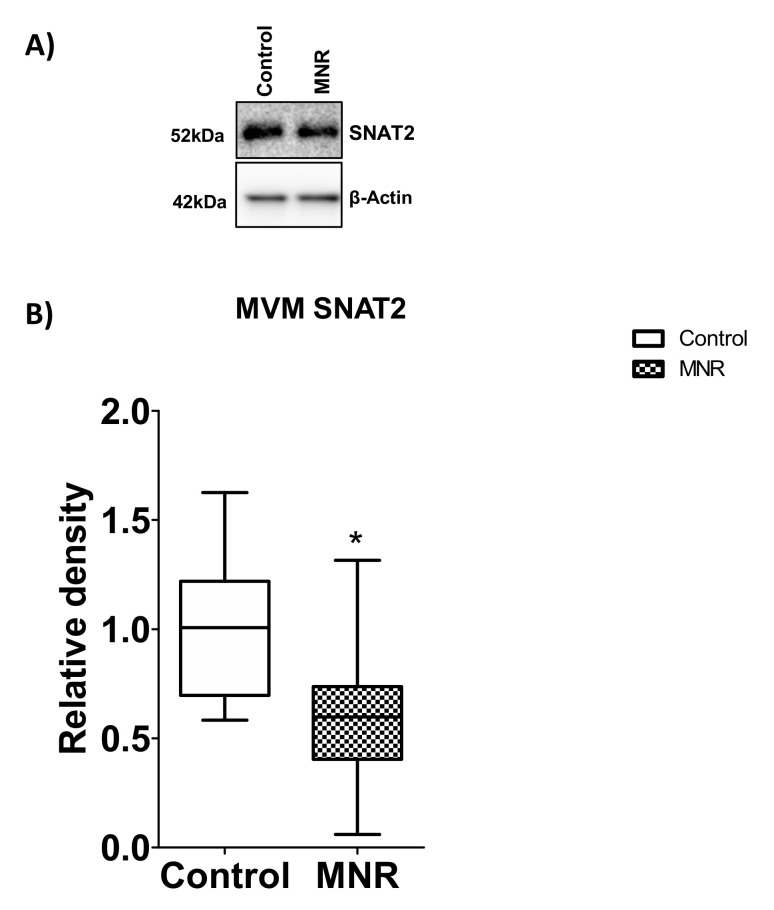
MVM SNAT2 protein expression at GD 140. (**A**) Western blot analysis of SNAT2 (system A amino acid transporter isoforms) expression in MVM isolated from control (C) and MNR (M) baboons. (**B**) The histogram shows protein expression data of MVM SNAT2. An equal amount of protein loading was performed. After normalization of MVM SNAT2 expression to loading control (β-actin), the average density of the control sample band was designated an arbitrary value of 1. Values are represented as Medians ± IQR. * *p* < 0.05, Mann–Whitney test (*n* = 8/each group). KS normality test *p*-value: MVM SNAT2 (Control, *p* > 0.10; MNR, *p* > 0.10; passed normality).

Next, we measured the SNAT2 protein expression in MVMs of the control and MNR group. Western blot analysis of SNAT2 expression in MVMs showed that SNAT2 expression was reduced by 41% (*p* = 0.04) in the MNR compared to controls at GD140 (Figure 9).

### 3.8. Protein Expression of TAUT and GLUT1 Transporter Isoforms in MVM at GD 140

**Figure 10:**
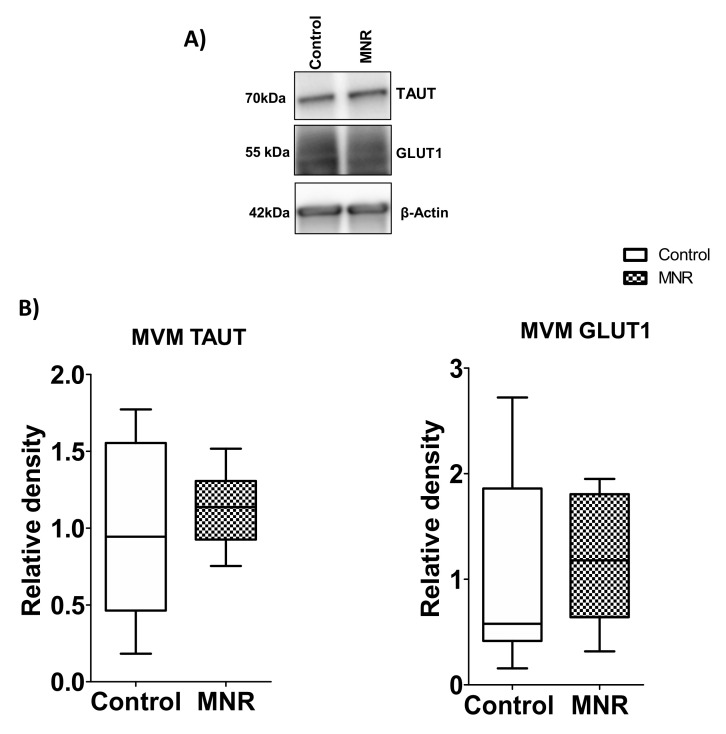
MVM TAUT and GLUT1 protein expression. (**A**) Western blot analysis of amino acid transporter isoforms TAUT and glucose transporter (GLUT1) in MVM isolated from control (C) and MNR (M) baboons at GD 140. (**B**) The histogram shows protein expression data of MVM TAUT and GLUT1. An equal amount of protein loading was performed. After the normalization of MVM TAUT and GLUT1 expression to loading control (β-actin), the average density of the control sample band was designated an arbitrary value of 1. Values are represented as Medians ± IQR. *n* = 9/each group. KS normality test *p*-value: MVM TAUT (Control, *p* > 0.10; MNR, *p* > 0.10; passed normality); MVM GLUT1 (Control, *p* = 0.04, not passed normality; MNR, *p* > 0.10; passed normality).

The MVM protein expression of the taurine transporter (TAUT) and glucose transporter (GLUT1) were determined in control and MNR baboons (Figure 10). The TAUT and GLUT1 protein expression in the MVM was not different between the control and MNR groups (TAUT, *n* = 9/each group, *p* = 0.78; GLUT1, *n* = 8/each group).

Significant changes in the fetal and placental weights, MVM and BM system A/L amino acid transport activity and isoform expression, maternal, fetal amino acid concentration, and in vivo transplacental amnio acid transport activity in MNR baboons as compared to control at GD 120 [34], GD 140 (current study) and GD 165 [11,38] are summarized in Table 2.

## 4. Discussion

Because of the inaccessibility of the human placenta for detailed functional studies before delivery, we used a well-characterized IUGR model in the baboon involving maternal nutrient restriction to induce fetal growth restriction to test the hypothesis that a reduction in the capacity of the placenta to transport amino acids precedes the development of fetal growth restriction. At GD 140, despite normal fetal growth, we found a robust reduction in the in vitro activity of two critical placental amino acid transporters, system A and L, and a reduction of tryptophan transfer in vivo in the MNR baboon. Studies on sonographic biometric measurements demonstrate that fetal and placental growth in baboons and humans is similar during mid and late gestation [39].

Together with our previous reports on placental amino acid transport in MNR at GD 120 [34] and 165 [38], this data allows us to construct a time course of nutrient transporter activity changes across the second half of pregnancy in response to MNR (Table 2). Our data suggest that down-regulation of placental amino acid transport in response to MNR directly contributes to restricted fetal growth in this maternal nutrition restriction model. Our findings are not consistent with the theory of compensatory up-regulation of placental nutrient transfer to maintain fetal development in response to restricted maternal nutrition [40]. These findings have important implications for our understanding of the pathophysiology of restricted fetal growth and for developing effective intervention strategies in IUGR.

System A transporter activity [22] and SNAT 2 protein expression are reduced in human idiopathic IUGR [18], and MVM System A activity has been reported to be associated with the severity of IUGR [21]. Furthermore, MVM SNAT2 expression is positively correlated with per gram of fetal and placental weight in human IUGR [18,23]. This data suggests that reduced amino acid concentrations [18,38] and decreased system A activity [18,34] in IUGR pregnancy may be due to down-regulated placental SNAT2 expression. These observations in humans are consistent with an essential role for placental System A mediated transport in determining fetal growth trajectories. In the current study, System A activity and SNAT 2 protein expression in MVM was reduced in MNR baboons at GD 140, when fetal growth remained unaffected. A mechanistic link between placental System A mediated amino acid transport and fetal growth is supported by recent studies in mice demonstrating that the trophoblast specific knockdown of System A amino acid transporter isoform is associated with IUGR [17]. Furthermore, MVM and BM system L amino acid transporter activity was reduced in MNR at GD 140, consistent with our previous reports that placental System L activity is decreased in human IUGR placentas [41].

The Fv/M MPE ratio of tryptophan was decreased and positively correlated with birthweight in MNR Baboons at GD140. This data suggested reduced capacity for transplacental transfer of tryptophan in response to MNR. MVM uptake of tryptophan is exclusively mediated via system L transport [42,43], whereas basal plasma membrane transport of tryptophan is mediated by system L and system y^+^L [43]. It is known that fetal tryptophan depletion impairs fetal cerebral 5-HT synthesis, with negative consequences for brain development [44]. In addition, tryptophan has a vital role in antioxidant activity [45]. It is also necessary for the formation of kynurenic acid, a neuroactive metabolite known to protect from hyperexcitability and anxiety, and an increase in its availability to the fetus is essential [44]. Therefore, reduced transplacental tryptophan transfer may contribute to disrupted neurodevelopment and high rates of aggressive behavior reported in 4-year-old IUGR baboons [46].

The Fv/M MPE enrichment ratio of leucine was positively correlated to the fetal weight in both control and MNR pregnancies. This finding agrees with previous studies demonstrating an impaired leucine flux in human IUGR and that the degree of change in leucine flux was correlated with the clinical severity of IUGR at term [41]. Leucine supplementation has been shown to activate the intracellular mTOR signaling pathways and prevent most growth-related deficits in rats exposed to a low protein diet during pregnancy [47]. Furthermore, the Fv/M MPE ratios for isoleucine, methionine, and histidine were positively correlated to fetal weight in MNR pregnancies, showing that the reduced MVM system A and system L activity contributes to decreased transplacental amino acid transport, which may lead to reduced amino acid concentrations in the fetal circulation. However, we did not find a decrease in Fv/M MPE enrichment ratio of most essential amino acids (except for tryptophan) in MNR despite significant changes in in vitro activity of MVM System A and L transport at GD140. The mechanisms underpinning this discrepancy may include decreased placental utilization of amino acids mediated by decreased placental protein synthesis and/or amino acid metabolism in MNR at GD 140, which may maintain in vivo transplacental transport even when transport capacity per membrane area (as measured in vitro) is reduced.

Maternal and fetal total amino acid concentrations were not measured in the current study. However, we have previously reported maternal and fetal amino acid concentrations in this model at GD 120 [34] and GD 165 [11]. These data show that at GD 120, the fetal and maternal amino acid concentrations are strikingly similar between the control and MNR groups, with no significant differences in maternal concentrations between groups. In addition, the levels of only two amino acids (leucine and isoleucine) were lower in the fetal plasma of MNR animals at GD 120. Similarly, at GD 165, the levels of only a few amino acids are lower in the maternal and fetal circulations, respectively. Because GD 120 and 140 have unaffected fetal growth in common, we suggest that it is likely that maternal and fetal amino acid concentrations at GD 140 are also similar between the control and MNR groups. Under these circumstances, relative differences in Fv/M MPE between the control and MNR groups provide information on the placental amino acid transfer.

The data presented in the current study, together with our previous reports on placental amino acid transport in the MNR baboon at GD120 [34] and GD 165 [38], allows us to construct a time course of changes in nutrient transporter activity across the second half of pregnancy in response to MNR in non-human primates (Table 2). In addition, this data addresses the question of cause-and-effect because although fetal weights were not significantly reduced until GD 165, MVM System A activity was decreased in response to MNR already at GD120, before decreased fetal weights were apparent. Moreover, at GD 140, with fetal weight unaffected, both MVM System A and L activity were lower in MNR placentas. This pattern of change in placental amino acid transport function across the third trimester in relation to the effect of MNR on fetal weights is consistent with the model that down-regulation of placental amino acid transport in response to MNR directly contributes to the restricted fetal growth.

**Figure 11:**
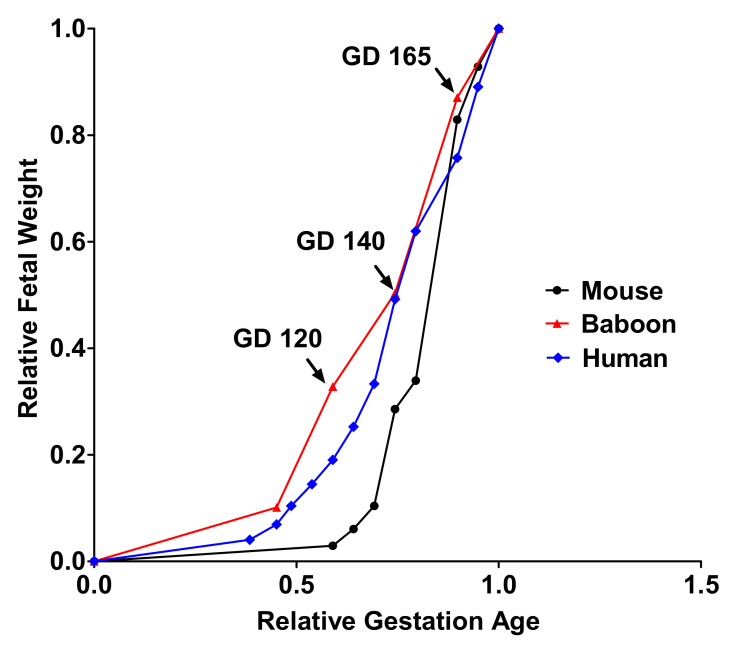
A timeline graph showing fetal growth curves for mice, baboons, and humans.

Establishing the time course of changes in placental amino acid transport across the last third of gestation will also allow us to identify compensatory changes in placental acid transport function that may occur in response to MNR. However, at no stage of late gestation could a compensatory up-regulation of placental System A and L amino acid transporter activity be observed (Table 2). A timeline graph showing fetal growth curves of mouse and human pregnancy describes how the current study time point corresponds to human and mice pregnancy (Figure 11).

Similarly, the MVM protein expression of GLUT 1, the primary placental glucose transporter in primates, and the taurine transporter were unaffected by MNR at GD 140 in the current study. Thus, although the effect of maternal undernutrition on placental function in animal models appears to depend on the species under investigation and the timing, duration, type, and degree of nutrient restriction [48], these findings are in general agreement with studies of calorie restriction in rats [49,50,51,52]. Similarly, in protein-restricted pregnant rats, a down-regulation of placental amino acid transport preceded IUGR by several days without evidence of compensatory up-regulation [9,12].

In some studies in mice, evidence for compensatory up-regulation of placental nutrient transporters in response to maternal undernutrition [53,54,55] has been reported. The current study suggests that placental nutrient transport in response to maternal nutrient deprivation is regulated differently in the non-human primate compared to the mouse. As discussed elsewhere [48], the distinct placental responses to maternal under-nutrition in the mouse as compared to the rat and the non-human primate may reflect actual species differences, differences in feeding paradigms, or methodology to measure placental transporter activity. These studies in the mouse have led to the proposal that fetal demand signals promote compensatory placental changes, such as up-regulation of placental amino acid transporters, in response to maternal undernutrition. However, although compensatory upregulation prior to GD 120 cannot be excluded, this model is not supported by our data in the baboon.

The small sample size, typical for non-human primates studies, limits our ability to perform more sophisticated statistical analyses and precludes detailed analyses of possible sex differences. Another limitation of our study is that the total concentrations of individual amino acids in the maternal and fetal circulations have not been measured.

## 5. Conclusions

We report that reducing placental amino acid transport precedes the development of fetal growth restriction in an established baboon model with extensive similarities to the human IUGR. Therefore, maternal supplementation with amino acids could be an option to increase fetal growth in IUGR pregnancies. However, it is essential to thoroughly understand the mechanisms of transplacental transport of amino acids and the impact of individual amino acids on placental metabolism and fetal growth before designing treatments for IUGR. This study represents the first step in understanding transplacental amino acid flux in IUGR in a non-human primate model, which shows extensive similarities to the human IUGR.

## Figures and Tables

**Table 1:** Composition of the infusate.

Amino Acid	Isotopic Labelling	Concentration of Amino Acid (mg/mL)	Molar Concentration
Valine	(1-^13^C)	1.25	0.01
Leucine	(1-^13^C)	1.25	0.009
Isoleucine	(1-^13^C)	1.25	0.009
Methionine	[Methyl-^2^H_3_]	1.25	0.008
Threonine	[U-^13^C_4_]	2.5	0.02
Phenylalanine	[1-^13^C]	1.25	0.007
Lysine	[4,4,5,5-^2^H_4_]	2.5	0.011
Histidine	[Ring 2-^13^C]	2.5	0.012
Tryptophan	[Indole-^2^H_5_]	2.5	0.012

All nine stable isotope-labeled (^13^C or ^2^H) essential amino acids were dissolved in PBS (physiological saline solution) [35].

**Table 2:** Comparison of significant changes in the fetal and placental weights, MVM and BM system A/L amino acid transport activity and isoform expression, maternal, fetal amino acid concentration, and in vivo transplacental amnio acid transport activity in MNR baboons as compared to control at different gestation ages.

Parameters	Gestation Day 120	Gestation Day 140	Gestation Day 165
Reference	[34]	Current study	[11,38]
Fetal weight	⬌	⬌	↓
Placental weight	⬌	⬌	↓
MVM System A activity	↓	↓	↓
MVM System A amino acid transporter isoforms (SNAT1,2 and 4) expression	⬌	↓SNAT2	↓SNAT2; ⬌ SNAT1, SNAT4
MVM System L activity	⬌	↓	↓
MVM System L amino acid transporter isoforms expression (LAT1 and 2)	⬌	↓LAT1	↓LAT1 and LAT2
MVM Taurine transporter expression	⬌	⬌	↓
BM System L activity	⬌	↓	↓
BM System L amino acid transporter LAT1 isoform expression	⬌	Not studied	Not studied
Maternal plasma concentration of amino acids	⬌	Not studied	↓Aspartic acid, glutamic acid, tyrosine, tryptophan, phenylalanine, leucine, and ornithine; ↑Glycine
Fetal plasma concentration of amino acids	↓ Leucine and isoleucine. ↑Citrulline	Not studied	↓Taurine, tyrosine, phenylalanine, leucine, and ornithine
In vivo transplacental amino acid transport	Not studied	↓Tryptophan	↓Leucine, isoleucine, methionine, phenylalanine, threonine, and tryptophan

## Data Availability

Not applicable.

## Supplementary Materials

The following are available online at https://www.mdpi.com/article/10.3390/nu13082892/s1, Figure S1: Relationship between MVM system A and system L activity at GD 140 (A). MVM system A activity was positively correlated to MVM system A activity in both control and MNR groups (control, r = 0.72, p = 0.02; MNR, r = 0.93, p = 0.0002, n = 9 in each group, Pearson correlation coefficient (r)). Figure S2: Relationship between MVM LAT1 and LAT2 expression at GD 140. MVM LAT1 expression was positively correlated to MVM LAT2 expression in the control group but not the MNR group (control, r = 0.69, p = 0.04; MNR, r = 0.64, p = 0.06, n = 9 in each group, r = Pearson correlation coefficient). Supplemental Methods.
