# Supplementary material for: Reduction of In Vivo Placental Amino Acid Transport Precedes the Development of Intrauterine Growth Restriction in the Non-Human Primate
Source: Nutrients. 2021 Aug 23;13(8):2892. doi: 10.3390/nu13082892 (PMC8401823; doi:10.3390/nu13082892)
Supplement: Supplementary file 1 [file nutrients-13-02892-s001.zip › nutrients-1335624-supplementary.pdf]

Supplemental Methods

Animals:

Female baboons were housed in groups of 10–16 with a single male. All the animals were housed in outdoor metal and concrete cages at the Southwest National Primate Research Center, San Antonio, Texas (35, 36). Housing baboons in an outdoor group social environment allows group social interaction and physical activity. The body weight was recorded as the mean of fifty individual measurements over thirty seconds.

Stable isotope-labeled essential amino acids

The maternal femoral artery catheter was used to obtain blood samples immediately before the infusion and then subsequently at ∼2-min intervals for up to 14 min after completing the EAA infusion. All blood samples were centrifuged at 2,500 g for 15 min at room temperature, and plasma was collected and immediately stored at −80°C until further analysis. Stable isotopic enrichments were analyzed with a gas chromatograph-mass spectrometer in the electron impact mode described elsewhere (1). Peaks were read at mass-to-charge ratios (m/z) 289/288 for L-[1-^{13}C] Valine, at m/z 303/302 for L-[1-^{13}C] Leucine and L-[1-^{13}C] Isoleucine, at m/z 337/336 for L-[1-^{13}C] Phenylalanine, at m/z 323/320 for L-[methyl-$^{2}H_3$] Methionine, at m/z 435/431 for L-[4,4,5,5-$^{2}H_4$] Lysine, at m/z 408/404 for L-[U-$^{13}C_4$] Threonine, at m/z 441/440 for L-[ring 2-^{13}C] Histidine, and at m/z 380/375 for L-[indole- $^{2}H_5$] Tryptophan.

The isotopic enrichment of EAA in maternal and fetal plasma is expressed as a mole percent excess (MPE). It is defined as a quantitative measure of the concentration of a stable isotope as a percentage of all isotopes, over and above its usual occurrence in nature (2). MPE of each amino acid was determined in maternal and fetal plasma. The fetal umbilical vein/maternal artery (Fv/M) MPE was measured as the ratio between fetal plasma amino acid enrichment over maternal plasma amino acid enrichment at the time of umbilical blood sampling (1, 3). The F/M MPE ratio represents the transport rates at a non-steady state for the individual EAAs. Comparing F/M MPE ratios between control and MNR groups provides information on relative differences in transport rates. Because the fetus cannot synthesize essential amino acids, and protein degradation is assumed to be small or negligible during the course of the experiment, the F/M MPE will be between 0 (no transport at all) and 1 (very rapid transport, where fetal MPE will reach maternal MPEs).

Placental MVM and BM Vesicles preparation

After initial centrifugation and Mg^{2+} precipitation steps, BM aggregated with Mg^{2+} were separated from MVM and purified on a sucrose gradient. MVM and BM preparations were snap-frozen in liquid nitrogen and stored at −80°C until further use. Alkaline phosphatase activity in the MVM and homogenate was measured according to standard methods. MVM enrichment was calculated using the MVM to homogenate ratio of alkaline phosphatase activity. MVM vesicle enrichment of alkaline phosphatase activity was comparable between the control and MNR groups (control, 7.7 ± 0.6, n = 8 vs. MNR, 7.9 ± 0.9, n = 8;). We have previously shown that the insulin receptor is highly expressed in the MVM of the baboon placenta (4). Therefore, the insulin receptor's protein expression in the MVM was analyzed using the Western blot as an additional enrichment marker. MVM enrichment of the insulin receptor in control (4.34 ± 0.57-fold, n = 6) and MNR groups (4.60 ± 0.52-fold, n = 9) was not significantly different. The protein expression of ferroprotein, an iron transporter localized almost exclusively to the BM (5) was determined using Western blot, and the BM/homogenate-ratio of ferroprotein expression was used as a measurement of BM enrichment. BM enrichment of ferroprotein was not significantly different between control and MNR groups (control, 21 ± 6.5, n = 6 vs. MNR, 24 ± 7.7, n = 6).

Activity of Amino Acid Transporters in MVM and BM

System A activity establishes the high intracellular concentration of non-essential amino acids, which are used to exchange for extracellular essential amino acids via System L. Thus, System A activity is critical for cellular uptake of both non-essential and essential amino acids. Thus, we measured both system A and L activity in MVM.

System A transporter activity was assessed in MVM by measuring the uptake of the non-metabolizable amino acid analog methylaminoisobutyric acid (MeAIB) using a slightly modified protocol as described previously (34). System L activity in the MVM and mediated leucine transport in the BM was measured by determining the transport of L-leucine (34, 35). The rationale to measure system L activity in BM is that the efflux of essential amino acids from the syncytiotrophoblast across the BM into the fetal circulation is mediated by System L transporters (41).

Briefly, vesicles were preloaded and incubated overnight in 300 mmol/L mannitol and 10 mmol/L HEPES-Tris, pH 7.4, at 4°C. Vesicles were subsequently centrifuged and resulting pellet was resuspended in the same buffer at a protein concentration of approximately 6 mg/ml. Vesicles were kept on ice until immediately prior to uptake measurements, when samples were warmed to 37°C using a water bath. Vesicles were mixed rapidly with 30 µl of the appropriate incubation buffer (1:2) including ^{14}C- MeAIB or ^{3}H-L-leucine as described previously (35, 36). After defined incubation times, vesicles were separated from the substrate medium using rapid filtration over mixed ester filters (0.45 µm pore size; Millipore Corporation) and washed using three times with 2 ml PBS. In studies of System A activity, 150 mmol/L NaCl and 150 mmol/L KCl were used in incubation buffers to measure total and Na^{+}-independent MeAIB uptake, respectively. In leucine transport experiments, nonmedicated flux was determined in the presence of 20 mmol/L unlabeled L-leucine. Each condition was measured in triplicate for each placenta in all uptake experiments as shown previously (35, 36).

Western Blot Analysis

Briefly, MVM samples (10 µg of total protein) were resolved onto a NuPAGE Novex (Invitrogen, Carlsbad, CA, USA) precast 4–12% Bis-Tris gels at 200 V for 40 min. Subsequently, proteins were transferred onto nitrocellulose membranes at 40 V for 1 hr. Membranes were blocked with blocking buffer (5% milk in Tris-buffered saline (w/v) plus 0.1% Tween 20 (TBS-T)) for one h at room temperature. Subsequently, membranes were probed with respective primary antibodies overnight at 4°C. After washing with TBS-T, membranes were probed with the appropriate peroxidase-labeled secondary antibodies for one hour. For detection of immunoreactive bands, blots were incubated with enhanced chemiluminescence detection reagents (Pierce Biotechnology, Rockford, IL, USA) and visualized using G Box. After stripping with stripping buffer, blots were re-probed for β-actin as a loading control. Density analysis of the immunoblot was performed using the G Box software.

References

1. Paolini, C. L., Meschia, G., Fennessey, P. V., Pike, A. W., Teng, C., Battaglia, F. C., and Wilkening, R. B. (2001) An in vivo study of ovine placental transport of essential amino acids. *Am J Physiol Endocrinol Metab* **280**, E31-39
2. Kim, I. Y., Suh, S. H., Lee, I. K., and Wolfe, R. R. (2016) Applications of stable, nonradioactive isotope tracers in in vivo human metabolic research. *Exp Mol Med* **48**, e203
3. Galan, H. L., Marconi, A. M., Paolini, C. L., Cheung, A., and Battaglia, F. C. (2009) The transplacental transport of essential amino acids in uncomplicated human pregnancies. *Am J Obstet Gynecol* **200**, 91 e91-97
4. Kavitha, J. V., Rosario, F. J., Nijland, M. J., McDonald, T. J., Wu, G., Kanai, Y., Powell, T. L., Nathanielsz, P. W., and Jansson, T. (2014) Down-regulation of placental mTOR, insulin/IGF-I signaling, and nutrient transporters in response to maternal nutrient restriction in the baboon. *FASEB J* **28**, 1294-1305
5. Bastin, J., Drakesmith, H., Rees, M., Sargent, I., and Townsend, A. (2006) Localisation of proteins of iron metabolism in the human placenta and liver. *Br J Haematol* **134**, 532-543

A)

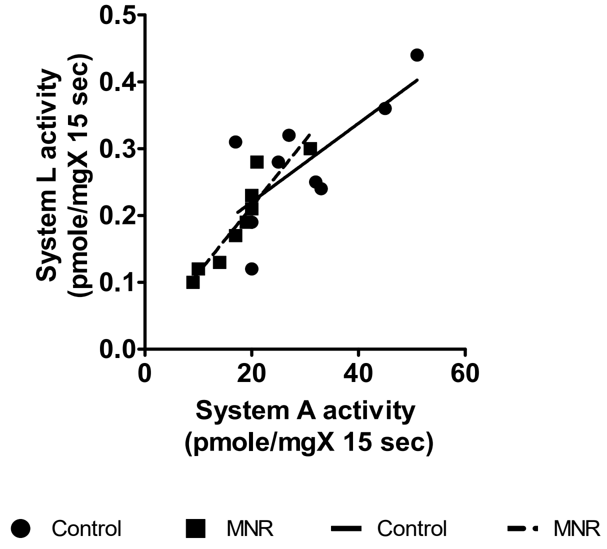

Supplemental Figure 1: Relationship between MVM system A and system L activity at GD 140 (A). MVM system A activity was positively correlated to MVM system A activity in both control and MNR groups (control, r = 0.72, P = 0.02; MNR, r = 0.93, P = 0.0002, n = 9 in each group, Pearson correlation coefficient (r)).

A)

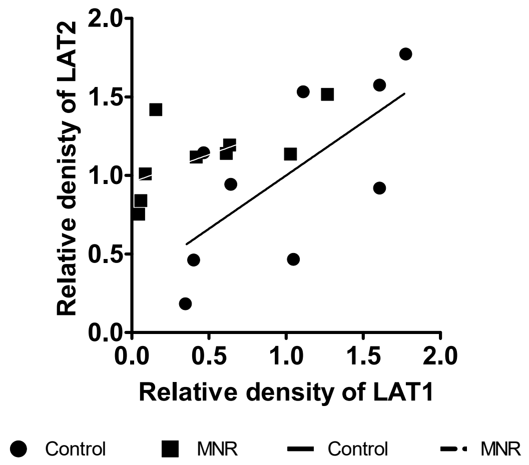

Supplemental Figure 2. Relationship between MVM LAT1 and LAT2 expression at GD 140. MVM LAT1 expression was positively correlated to MVM LAT2 expression in the control group but not the MNR group (control, r = 0.69, P = 0.04; MNR, r = 0.64, P = 0.06, n = 9 in each group, r = Pearson correlation coefficient).
